# Supplementary material for: Effect of Therapeutic Gardening Program in Urban Gardens on the Mental Health of Children and Their Caregivers with Atopic Dermatitis
Source: Healthcare (Basel). 2024 Apr 29;12(9):919. doi: 10.3390/healthcare12090919 (PMC11083003; doi:10.3390/healthcare12090919)
Supplement: Supplementary file 1 [file healthcare-12-00919-s001.zip › healthcare-2930414-supplementary.pdf]

healthcare

MDPI

Supplementary Tables & Figure

Table S1. Measurement tools used.

Classification	Measurement
Psychosocial effect measurement	Korean Version of Mental Health Screening Tool: Depressive Disorders (MHS:D)<br>Korean Version of Mental Health Screening Tool: Anxiety Disorders (MHS:A)<br>Korean version of Core life activities index (CORE)<br>Korean version of the Satisfaction with the Life Scale (K-SWLS)<br>Korean version of Parenting Self-Efficacy (PSE)<br>Korean version of Perceived Stress Scale (PSS)
	Children's Depression Inventory (CDI)
Physiological effect measurement	SCORing Atopic Dermatitis Index (SCORAD Index)
	Transepidermal water loss (TEWL)

Table S2. Therapeutic garden program (15 sessions).

Step	Session	Program theme	Garden activities
Garden for Start	1	Introduction	Orientation: Introducing myself with plants, guide to research contents and program, notices, etc.
	2	Setting up garden 1	Making bed, sowing seeds, checking soil composition and Blending soil
	3	Setting up garden 2	Introducing the plants in the garden and understanding their characteristics Planting
Garden for Participation	4	Garden design	Planting herbs & edible plants for stress and psychological stability, creating herb garden that harmonizes with flowers, understanding companion plants
	5	Garden maintenance	Learning the basics of gardening: watering, fertilizing, composting, fielding & erecting poles
	6	Garden design	Making seasonal garden: Garden design & planting plan of various colors to feel the change of the season
	7	Garden maintenance	Managing weeds and pests: making & spaying natural insect repellent, pesticide
Garden for Experience	8	Garden Utilization	Using natural herbs: making herbal tea and herbal soap using herbs harvested from the garden
	9	Garden design	Making grass & rock garden: Stimulate the sense of touch by planting plants of various textures and experience plants growing in various environments.
	10	Garden Utilization	Cottage & division: learning and practicing various plant propagation methods (unsexual propagation, sexual propagation)
	11	Garden Cultural Activities	A family program where you spend time in the garden: making a birdhouse, bouquets, treasure hunt in the garden, etc.
Garden for Restart	12	Garden Utilization	Harvesting summer & fall flower and gathering seed, caring for garden of next year
	13	Garden Cultural Activities	Sharing the plants and collected seeds from the last program
	14	Garden maintenance	Garden planning: Detailed planning for the next garden with gardening calendar, planting bulbs for next spring, preparing for the coming winter
	15	Garden Cultural Activities	Garden party with photography exhibition

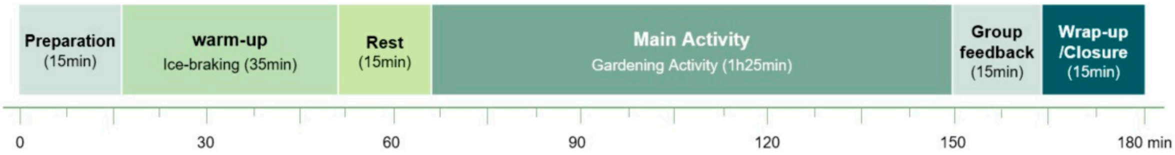

Figure S1. Timeline (in minutes) of program.

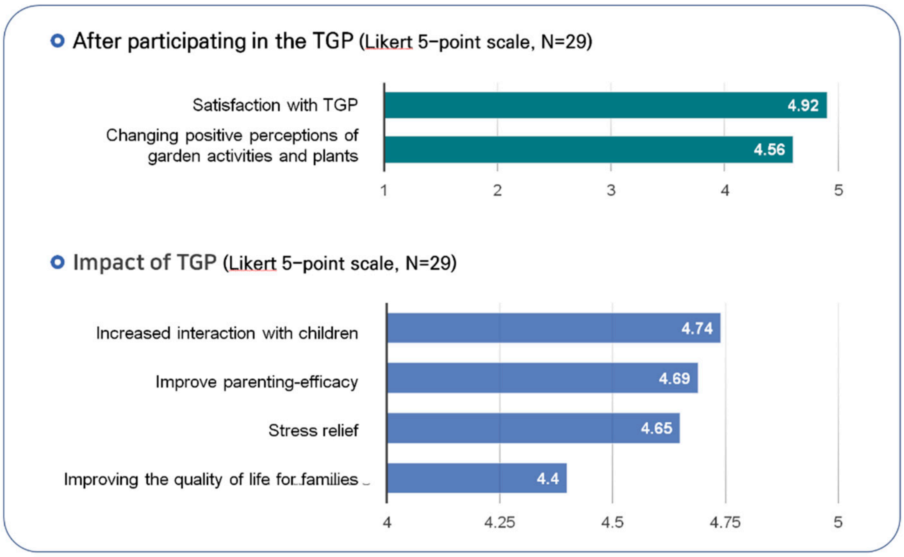

Figure S2. Result of therapeutic gardening program (TGP) satisfaction survey.
